# Correction: Evaluation of anxiety level and the factors Affecting Anxiety in health care workers in Shahid Dr. Gholipour Hospital, Bukan, Iran during COVID-19 pandemic

**DOI:** 10.1371/journal.pone.0321236

**Published:** 2025-03-27

**Authors:** Esmaiel Maghsoodi, Edris Hasanpour, Farzaneh Soleimani, Moosa Aghal, Keyvan Mollarahimi

There are errors in the author affiliations. The correct affiliations are as follows:

Esmaiel Maghsoodi^1^, Edris Hasanpour^1^, Farzaneh Soleimani^2^, Moosa Aghal^2^, Keyvan Mollarahimi^3^

1 Research Center for Evidence-Based Health Management, Maragheh University of Medical Sciences, Maragheh, Iran, 2 Department of Nursing, Maragheh University of Medical Sciences, Maragheh, Iran, 3 Department of Nursing, Boukan Nursing School, Urmia University of Medical Sciences, Urmia, Iran.
