# Supplementary material for: Evaluation of anxiety level and the factors Affecting Anxiety in health care workers in Shahid Dr. Gholipour Hospital, Bukan, Iran during COVID-19 pandemic
Source: PLoS One. 2024 Dec 13;19(12):e0264916. doi: 10.1371/journal.pone.0264916 (PMC11644838; doi:10.1371/journal.pone.0264916)
Supplement: Attachment — Submitted filename: comments.docx [file pone.0264916.s002.docx]

**Introduction,**

Line 5 in the introduction, what is the “O”

Line 11 in the introduction, check 41-9-45.7%

**Methodology,**

The design of the study is descriptive cross sectional not descriptive analytical

Line 7, it should be “participate” not “participant”

Regarding Spielberger questionnaire, where is the reference of the used questionnaire

“Validity and reliability of the questionnaire were examined by Mahran and P-value was obtained equal to 0.81 with confidence level of 95%.”. P-value is not correct in this sentence. It should be Cronbach’s alpha

**Results,**

Titles of the tables and figures are short and non-explanatory

Table (1): in education, the sum is 360 not 358. It should be 358

Table (4): the factors related to hidden or obvious anxiety??

Figure (1) is not clear at all

The results should include logistic regression analysis to determine the significant predictors of anxiety.

**Discussion,**

Deficient with no enough comparable results
