# Supplementary material for: Evaluation of anxiety level and the factors Affecting Anxiety in health care workers in Shahid Dr. Gholipour Hospital, Bukan, Iran during COVID-19 pandemic
Source: PLoS One. 2024 Dec 13;19(12):e0264916. doi: 10.1371/journal.pone.0264916 (PMC11644838; doi:10.1371/journal.pone.0264916)
Supplement: Attachment — Submitted filename: Response to Reviewers.docx [file pone.0264916.s005.docx]

Dear Dr. Tauqeer Hussain Mallhi:

Thank you for your attention, all issues raised in the final edition were corrected and resubmitted.

**Reviewer’s comments**

Title: **Examining anxiety level in medical team members in Shahid Dr. Gholipour Hospital, Bukan, Iran during COVID 19 pandemic**

Manuscript number: **PONE-D-20-38720**

**Corresponding Author:** keyavan mollararahimi Urmia University of Medical Sciences Urmia, IRAN, ISLAMIC REPUBLIC

**Comments:**

1. General comments: It is simple to comment if the manuscript had numbers on the lines of manuscript. Improve it. Another general comment is that just write affiliations of staffs in similar institutions by one sentence.
2. Page 3: It is better to improve title to “**Evaluation of** **anxiety level in medical team members in Shahid Dr. Gholipour Hospital, Bukan, Iran during COVID 19 pandemic** instead of verb Examining
3. Page 3: Under introduction of summary you indicated objective as “examine anxiety level and the factors in medical team members in Shahdi Dr. Gholipour Hospital, Bukan, Iran during Covid-19 pandemic.” This objective is different from title. So, include factors in medical team members to the research title
4. Page 3: In the methodolgy section please indicate analysis technique followed and revise methodology section that why you used census sampling technique. This will reduce generalizability of your findings. What type of descriptive analytical design you used? Indicate the specific study design.
5. Page 3: What is moderate to high level of anxiety among workers? Respond to ideas accordingly
6. Page 3: In the conclusion section please include major findings and factors determining anxiety level.
7. Page 4: Define anxiety at the start of the manuscript so that it makes readers familiar with the title from the start.
8. Page 5: Indicate type of specific study used in the study. This is critical and there are many descriptive analytical study designs. Choose one specific study design
9. Page 5: There are different study participants you selected from hospital. How you selected them. Please indicate sampling strategy you used specifically for this case. You described that census sampling strategy used. But many of study participants is not included if this is case. Justify it accordingly and it is critical issue.
10. Page 5: Do you think reluctance is right exclusion criteria. Revise clearly about study participants characteristics
11. Page 6: What of statistical model used in identifying factors related to medical team workers. Please review and add methods of analysis used in the study
12. Page 6: Majority of results not reported. Please report socio-demographic variables separately and indicate study response rate. Please rewrite results and report findings only. Line 183 there are interpretation of results
13. Page 7: How you identified relations between variables. How you identified model fitness in this study. You have three dependent variable and how analyzed them while identifying factors related to it.
14. Page 7: Discussion is too short and discuss every factors related to anxiety level and indicate reason for difference or similarity
15. Page 7: Too narrow conclusion and include all issues identified while recommending stakeholders
16. Page 7: Revise manuscript based on PLOS ONE guideline. Please indicate abbreviations’ used in the study

**Chief editor**

**Introduction,**

Line 5 in the introduction, what is the “O”

Line 11 in the introduction, check 41-9-45.7%

**Methodology,**

The design of the study is descriptive cross sectional not descriptive analytical

Line 7, it should be “participate” not “participant”

Regarding Spielberger questionnaire, where is the reference of the used questionnaire

“Validity and reliability of the questionnaire were examined by Mahran and P-value was obtained equal to 0.81 with confidence level of 95%.”. P-value is not correct in this sentence. It should be Cronbach’s alpha

**Results,**

Titles of the tables and figures are short and non-explanatory

Table (1): in education, the sum is 360 not 358. It should be 358

Table (4): the factors related to hidden or obvious anxiety??

Figure (1) is not clear at all

The results should include logistic regression analysis to determine the significant predictors of anxiety.

**Discussion,**

Deficient with no enough comparable results

Reviewer #5: The authors should state the appropriate mode they used for their data analysis. If logistic regression analysis was used, how?
Method should be clearly stated.
Have you checked internal consistency?
How did you assess your outcome of interest to use logistic regression model?
What are assumptions of your appropriate model for data analysis?

Keyvan Mollarahimi

Corresponding Author

Kind regards
